# Characterization of Tumor Suppressive Function of *cornulin* in Esophageal Squamous Cell Carcinoma

**DOI:** 10.1371/journal.pone.0068838

**Published:** 2013-07-24

**Authors:** Kai Chen, Yan Li, Yongdong Dai, Jiangchao Li, Yanru Qin, Yinghui Zhu, Tingting Zeng, Xiaojiao Ban, Li Fu, Xin-Yuan Guan

**Affiliations:** 1 State Key Laboratory of Oncology in Southern China, Cancer Center, Sun Yat-Sen University, Guangzhou, China; 2 Department of Clinical Oncology, the University of Hong Kong, Hong Kong, China; 3 Department of Clinical Oncology, the First Affiliated Hospital, Zhengzhou University, Zhengzhou, China; 4 Vascular Biology Research Institute, Guangdong Pharmaceutical University, Guangzhou, China; Vanderbilt University Medical Center, United States of America

## Abstract

By using cDNA microarray analysis, we identified cornulin (*CRNN*) gene was frequently downregulated in esophageal squamous cell carcinoma (ESCC). In the present study, we investigated the role of *CRNN* in ESCC development. The results showed that CRNN was frequently downregulated in primary ESCCs in both mRNA level (26/56, 46.4%) and protein level (137/249, 55%), which was significantly associated with lymph node metastases (*P*=0.027), advanced clinical stage (*P*=0.039), and overall survival rate (*P*<0.001). Multivariate analysis indicated that the CRNN downregulation was an independent prognostic factor for ESCC. Functional studies with both *in vitro* and *in vivo* assays demonstrated that CRNN had strong tumor suppressive ability in ESCC cells. The tumor-suppressive mechanism of CRNN was associated with its role in cell cycle arrest at G1/S checkpoint by upregulating expressions of P21^WAF1/CIP1^ and Rb. Silencing *CRNN* expression by RNA interference could effectively inhibit its tumor suppressive effect. In conclusion, our findings demonstrate that CRNN is a tumor suppressor gene that plays a critical tumor suppressive role in ESCC.

## Introduction

Esophageal carcinoma (EC) is one of the most common malignancies and has been ranked as the sixth leading cause of cancer death over the world [1]. As the most common type of EC, esophageal squamous cell carcinoma (ESCC) shows high mortality and regional variation in incidence [2]. Despite advances in multimodality therapy, the prognosis of ESCC remains poor and the overall 5-year survival is less than 15% [3]. Like other types of cancers, the development of ESCC is also believed as a multiple-step process caused by the accumulation of activation of oncogenes and inactivation of tumor suppressor genes (TSG). To date, the exact cellular and molecular mechanisms leading to ESCC have not been systematically evaluated.

Systematic analysis of expression levels of thousands of genes by cDNA microarray is an effective approach to identify new genes and pathways related to the development and progression of the tested cancer. Recently, our group performed an Affymetrix cDNA microarray to compare differentially expressed genes between 10 pairs of ESCC tumors and their adjacent non-tumorous tissues (Data have been submitted to Gene Expression Omnibus under the accession number GSE33810). About 220 downregulated genes were detected including cornulin (*CRNN*). *CRNN* gene comprises three exons and encodes a protein of 495 amino acids, which contains a putative calcium-binding motif similar to S100 protein family at N-terminus [4], implying that CRNN may bind to calcium. Another study demonstrates that CRNN, which is a member of the fused gene family, might play an important role in epidermal differentiation [5].

Although CRNN has been reported to be downregulated in esophageal adenocarcinoma (EAC) or ESCC [6–9], and genetic variants of *CRNN* appeared to interacte with tobacco smoking that contributes to the risk for ESCC [10], the precise mechanism underlying the involvement of CRNN in ESCC remains to be elucidated.

In the present study, we studied the expression status of CRNN in clinical ESCC specimens and ESCC cell lines by quantitative and semiquantitative RT-PCR respectively. Both *in vitro* and *in vivo* functional assays were used to investigate the tumor suppressive effect of CRNN in ESCC cell lines. The results demonstrated that CRNN had strong tumor suppressive function. In addition, the tumor suppressive mechanism of CRNN and its clinical significance in ESCC were also addressed.

## Materials and Methods

### Cell lines and primary ESCC specimens

Chinese ESCC cell lines HKESC1, EC18 and EC109 were kindly provided by Professor Srivastava (Department of Pathology, The University of Hong Kong) [11]. Six Japanese ESCC cell lines (KYSE30, KYSE140, KYSE180, KYSE410, KYSE510, KYSE520) were obtained from DSMZ, the German Resource Center for Biological Material [12]. Primary ESCC tumors and their adjacent non-tumorous tissues were collected immediately after surgical resection at Linzhou Cancer Hospital (Henan, China). All patients did not receive preoperative treatment. Samples used in this study were approved by the Committees for Ethical Review of Research involving Human Subjects at Zhengzhou University (Henan, China). Written informed consents for the original human work that produced the tissue samples were obtained. The study was also approved by the Institutional Review Board at Cancer Center, Sun Yat-sen University.

### Quantitative and semiquantitative RT-PCR

Total RNA was extracted by Trizol (Invitrogen, Carlsbad, CA) and 2µg of total RNA was used to synthesize cDNA with the Advantage RT-for-PCR Kit (Clontech, Mountain View, CA), following the standard protocols provided by the manufacturer. Semiquantitative RT-PCR was performed by using AmpliTaq (Applied Biosystems, Foster City, CA). The *GAPDH* or 18s were used as internal controls. For qRT-PCR, cDNA were amplified using a SYBR Green PCR Kit (Roche, Basel, Switzerland). The sequences of primers were listed in Table 1. Amplification protocol consisted of incubations at 95^°^C for 15sec, 60 ^°^C for 1min for 40 cycles. Quantification was done using the ABI PRISM 7900HT Sequence Detection System (Applied Biosystems, Foster City, CA). All gene expression values were normalized using the internal control and calculated using the comparative C_T_ method (ΔΔC_T_ method) [13]. Downregulation was determined if relative quantification (RQ) value of non-tumor tissue was more than 2-fold change than RQ of corresponding tumor tissue.

**Table 1:** Primers’ sequences.

**Primers**	**sequence**
*CRNN*- F	5’-ACTCTTGGAGCAAGAGTTTG-3’
*CRNN*- R	5’-TGGAGGCTTCCAGAACTCTTG-3’
*p53*-F	5’-TTGCCAACTGGCCAAGACCTG-3’
*p53*-R	5’-ACGCAAATTTCCTTCCACTCGG-3’
*p21*-F	5’-TGTCCGTCAGAACCCATGC-3’
*p21*-R	5’-AAAGTCGAAGTTCCATCGCTC-3’
*GAPDH*-F	5’-CATGAGAAGTATGACAACAGCCT-3’
*GAPDH*-R	5’-AGTCCTTCCACGATACCAAAGT-3’
*18s*-F	5’-CTCTTAGCTGAGTGTCCCGC-3’
*18s*-R	5’-CTGATCGTCTTCGAACCTCC-3’

### Tissue microarray (TMA) and immunohistochemistry (IHC)

A TMA composed of 300 ESCC tumor specimens were collected from Linzhou Cancer Hospital (Henan, China). Tissue samples used in this study were approved by the Committees for Ethical Review of Research Involving Human Subjects at Zhengzhou University. Written informed consents for the original human work that produced the tissue samples were obtained. TMA was constructed as described previously [14]. IHC staining was carried out following standard streptavidin-biotin-peroxidase complex method [15]. Briefly, TMA sections were deparaffinized, and nonspecific bindings were blocked with 10% normal goat serum for 10min. The TMA section was then incubated with anti-CRNN polyclonal antibody (1:100 dilution, Abcam, Cambridge, UK) at 4 ^°^C overnight. Slides were then incubated with HRP-conjugated goat anti-rabbit immunoglobulin at a concentration of 1:100 at 37^°^C for 30min. Cytoplasmic expression of CRNN was assessed by three independent investigators. The immunoreactivity of CRNN was scored by staining intensity only (0 = negative staining; 1 = weak staining; 2 = strong staining) because no obvious difference was observed in the percentage of cells stained.

### *In vitro* tumorigenic assays

To test tumor suppressive function of CRNN, *CRNN* was cloned into pcDNA3.1/V5-His TOPO TA vector (Invitrogen, Carlsbad, CA) and transfected into ESCC cell line KYSE30 and KYSE180 cells (CRNN-30 and CRNN-180, respectively). Stable CRNN-expressing clones were selected for further study. Empty vector-transfected KYSE30 and KYSE180 cells (Vec-30/Vec-180) were used as controls. Cell growth, foci formation, and soft agar assays were carried out as described previously [11]. For cell growth assay, 1×10^3^ cells were seeded into 96-well plate and cell growth rate was detected using cell proliferation XTT kit (Dojindo, Japan) according to the manufacturer’s instructions. For foci formation assay, 1×10^3^ cells were plated in wells of a 6-well plate. After 7 days culture, surviving colonies (> 50 cells/colony) were counted with crystal violet staining. For soft agar assay, 5×10^3^ cells were seeded into 0.4% bactoagar on a bottom layer of solidified 0.6% bactoagar in 6-well plates. After 3 weeks, colonies consisted of more than 80 cells were counted. All above assays’ data were expressed as the means ± S.E.M. of triplicate independent experiments.

### Tumor formation in nude mice

The study was approved by Institutional Animal Care and Use Committee of Cancer Cancer, Sun Yat-sen University. Animal experiments were performed in compliance with the guidelines for the Welfare of Experimental Animals in Cancer Center, Sun Yat-sen University. The *in vivo* tumor-suppressive ability of CRNN was investigated by tumor xenograft experiment. About 2×10^6^ CRNN-transfected cells and empty vector-transfected cells were injected subcutaneously into the right and left sides of 4-week-old nude mice (n=9 for KYSE30 and n=6 for KYSE180), respectively. Tumor formation in nude mice was monitored by measuring the tumor volume, which was calculated by the formula, V=0.5×L (length of tumor) ×W^2^ (width of tumor) over a 4-week period [16]. To confirm CRNN expression in xenograft tumor, IHC staining with anti-CRNN antibody was performed in sections (5µm in thick) of paraffin-embedded xenograft tumor.

### RNA interfering (RNAi)

CRNN-expressing clones CRNN-C2, C3 (KYSE30) or CRNN-C1 (KYSE180) were transfected with double-stranded siRNAs (Ambion, Carlsbad, CA) with lipofectamine 2000^TM^ reagent (Invitrogen) according to the manufacturer’s instructions. Forty-eight hours after transfection, the gene-silencing effect was measured by qRT-PCR and western blot analysis, respectively. Three independent experiments were performed.

### Cell cycle analysis

CRNN-C2/CRNN-C1 or Vec-30/Vec-180(2×10^5^) were fixed in 70% ethanol and stained with propidium iodide, and DNA content was analyzed by Cytomics FC (Beckman Coulter, Indianapolis, IN).

### Western blot analysis

Western blotting was done according to the standard protocol with antibodies for GAPDH, CRNN (Santa Cruz Biotechnology, Santa Cruz, CA), P53, P21^WAF1/CIP1^, cyclin D1, CDK4, cyclin E, CDK2, Rb, tubulin (Cell Signaling Technology, Danvers, MA).

### Statistical analysis

Statistical analysis was performed using SPSS standard version 13.0 software (SPSS Inc, Chicago, IL). Data were expressed as mean ± S.E.M. from at least three independent determinations. Significance of difference was analyzed using Student’s *t*-tests. The correlation between CRNN expression and clinicopathologic characteristics was analyzed using the Fisher’s exact test. Cum survival was calculated from the date of diagnosis to the date of cancer-related death or last follow-up. Survival curve was assessed by the Kaplan-Meier method and compared by the log-rank test. Relative risks of cancer-related death associated with CRNN expression status and other predictor variables were estimated by univariate analysis. Multivariate survival analysis was done on all parameters that were found to be significant on univariate level using the Cox regression model. Differences were considered significant for *P*<0.05.

## Results

### Downregulation of CRNN is frequently detected in ESCC

**Figure 1:**
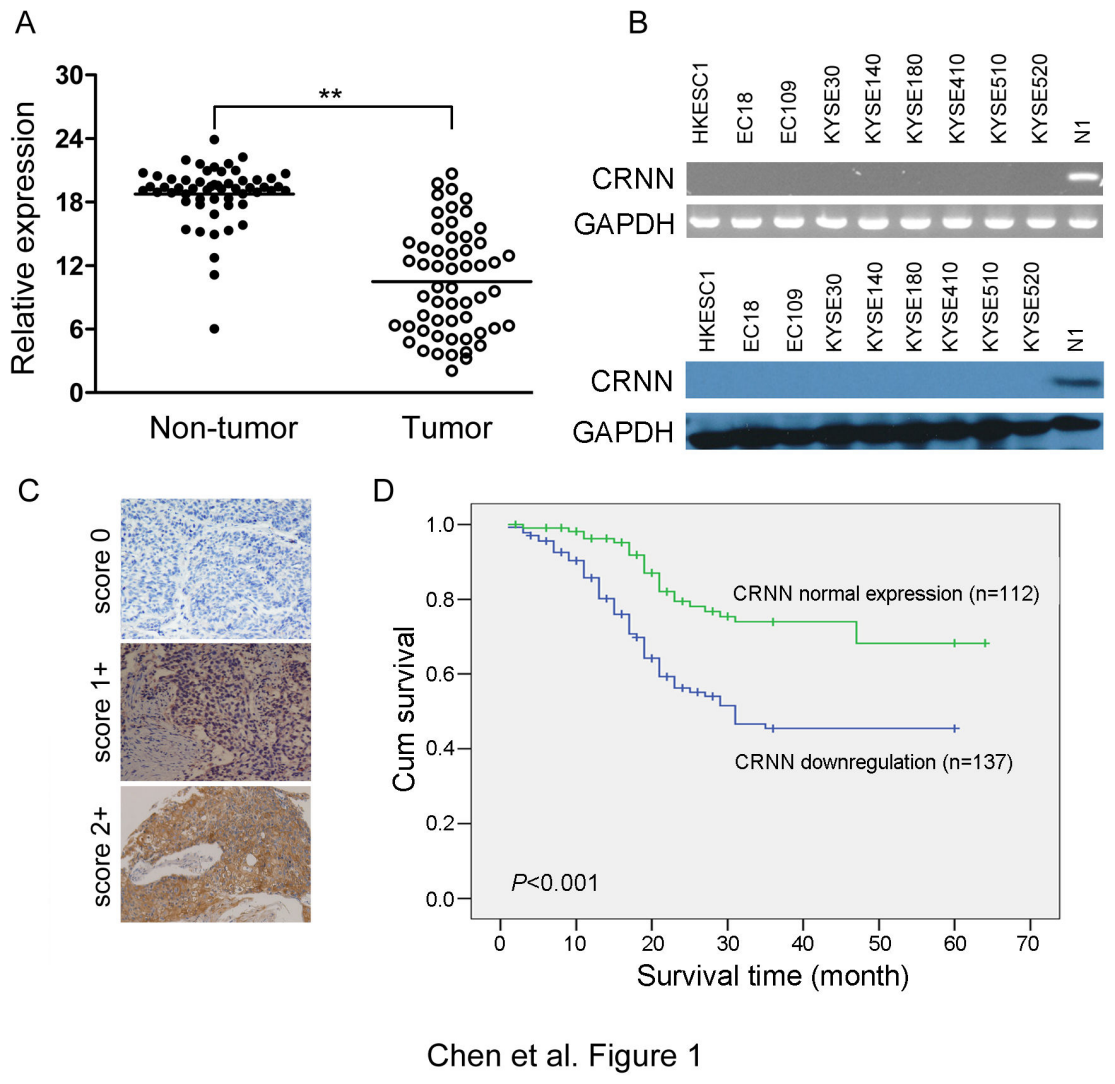
Downregulation of CRNN in ESCC. (A) qRT-PCR was used to compare CRNN mRNA levels between tumor and corresponding non-tumor tissues in 56 ESCC cases. **, *P*<0.01. (B) Absent expression of CRNN could be observed in all tested ESCC cell lines in both mRNA level (upper panel) and protein level (lower panel) detected by RT-PCR and western blot analysis, respectively. GAPDH was used as a loading control. N1, pool total RNA or protein from 5 non-tumor esophageal tissue specimens. (C) Representatives of immunostaining with anit-CRNN antibody in ESCC cases. Positive staining (brown) was detected in non-tumor esophageal epithelial cells but not in tumor cells. The slide was counterstained with hematoxylin. Original magnification, 200×magnification. (D) Kaplan-Meier analysis shows that downregulation of CRNN was significantly associated with poorer overall survival in 249 ESCC cases (*P*<0.001, Log-rank test).

The mRNA expression of CRNN in 9 ESCC cell lines and 56 primary ESCC tumors and their paired non-tumorous tissues were detected by semiquantitative and qRT-PCR, respectively. The results showed that downregulation of CRNN was detected in 26/56 (46.4%) of primary ESCCs (Figure 1A) and 9/9 of ESCC cell lines (Figure 1B).

### CRNN downregulation correlates with poor outcome of ESCC

To investigate the clinical significance of CRNN downregulation in esophageal carcinogenesis, CRNN expression in protein level was also studied using ESCC tissue microarray. Expression of CRNN was classified into absent (scored as 0), weak-positive (scored as 1+) and strong-positive (scored as 2+) cytoplasmic staining. Informative expression of CRNN was detected in 249 ESCC cases. Noninformative samples included lost sample and sample with too few tumor cells; such cases were excluded for data complication. Normal expression of CRNN (strong/weak staining) was observed in all non-tumorous esophageal epithelial cells (Figure 1C). Downregulated expression of CRNN (absent staining) was detected in 137/249 (55.02%) of informative ESCC cases.

The correlation of CRNN expression with various clinicopathologic features was investigated and the result showed that downregulation of CRNN was significantly associated with advanced clinical stage (*P*=0.039) and lymph node metastases (*P*=0.027, Table 2). Furthermore, log-rank test showed that ESCC patients with CRNN downregulation (mean survival time: 36 months) had a significant shorter survival time than patients with CRNN normal expression (mean survival time: 51 months; *P*<0.001) (Figure 1D). By univariable analyses, downregulation of CRNN (*P*<0.001), tumor differentiation (*P*=0.018), tumor invasion (*P*=0.007), and presence of lymph node metastases (*P*=0.001) were significantly negative prognostic factors for cum survival in ESCC patients (Table 3). Nevertheless, multivariable analyses showed that downregulation of CRNN and lymph node metastases were independent prognostic markers for ESCC patients enrolled in this study (*P*<0.05, Table 3).

**Table 2:** Association of CRNN downregulation with clinicopathological features in 249 ESCCs.

**Clinical features**	**Number**	**CRNN downregulation**	***P* value^#^**
***Age(yearsold)***			0.907
≤57	117	61 (52.1%)	
>57	132	76 (57.6%)	
***Gender***			0.873
Male	137	76 (55.5%)	
Female	112	61 (54.5%)	
***Differentiation***			0.243
Well	56	36 (64.3%)	
Moderate	162	88 (54.3%)	
Poor	31	13 (41.9%)	
***Tumorinvasion***			0.371
T_1_	15	7 (46.7%)	
T_2_	19	11 (57.9%)	
T_3_	54	24 (44.4%)	
T_4_	161	95 (59.0%)	
***Clinicalstage***			***0.039***
I	11	6 (54.5%)	
II	155	76 (49.0%)	
III	83	55 (66.3%)	
***Lymphnodemetastasis***			***0.027***
N_0_	141	69 (48.9%)	
N_1_	108	68 (63.0%)	

#: 2-tailed Fisher’s exact test.

**Table 3:** Cox proportional hazard regression analyses for overall survival.

**Clinical features**	Univariable analysis*		Multivariable analysis*	
	**HR (95% CI)**	***P* value**	**HR (95% CI)**	***P* value**
Gender	1.143 (0.740-1.764)	0.547		
Age	1.000 (0.977-1.025)	0.981		
Differentiation	1.565 (1.080-2.268)	***0.018***	1.408 (0.964-2.057)	0.077
Tumor invasion	1.526 (1.125-2.072)	***0.007***	1.412 (0.964-2.069)	0.077
LN metastasis	2.059 (1.338-3.168)	***0.001***	1.980 (1.017-3.856)	***0.045***
CRNN	0.375 (0.232-0.607)	***<0.001***	0.429 (0.263-0.701)	***0.001***

* HR = hazard ratio; CI = confidence interval.

### CRNN has strong tumor suppressive ability

**Figure 2:**
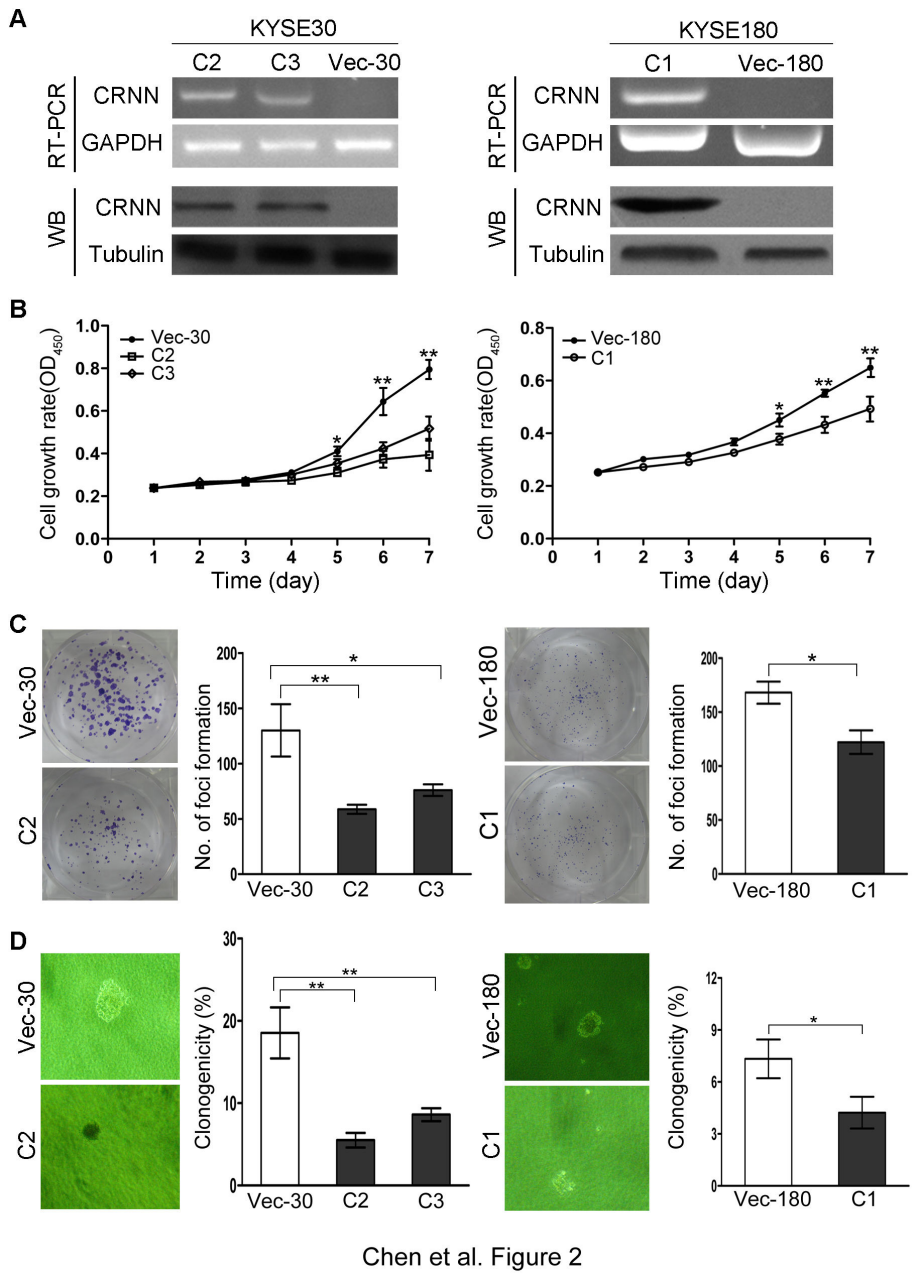
Tumor-suppressive function of CRNN in ESCC cell lines. (A) Expression of CRNN in CRNN-transfected ESCC cell lines KYSE30 (clones 2 and 3) and KYSE180 (clone 1) were confirmed by RT-PCR and western blot analysis, respectively. Empty vector-transfected ESCC cells (Vec-30 and Vec-180) were used as controls. GAPDH and Tubulin were used as internal and loading controls, respectively. (B) Growth curves of CRNN-transfected cells were compared with controls by XTT assay. Results were summarized from three independent experiments. *, *P*<0.05; **, *P*<0.01. (C) Representative inhibition of foci formation in monolayer culture by CRNN. Quantitative analyses of foci numbers are summarized from three independent experiments. *, *P*<0.05; **, *P*<0.01. (D) Inhibition of colony formation in soft agar by CRNN. The results are summarized from three independent experiments. *, *P*<0.05; **, *P*<0.01.

To determine if CRNN has tumor suppressive function, CRNN gene was stably transfected into ESCC cell lines KYSE30 and KYSE180 cells. Stably CRNN-expressing clones from KYSE30 (CRNN-C2 and CRNN-C3) and from KYSE180 (CRNN-C1) were selected. Empty vector-transfected cells (Vec-30 and Vec-180) were used as controls. Expression of CRNN in these clones was confirmed by RT-PCR and western blotting (Figure 2A). Tumor suppressive function of CRNN was assessed by cell growth, foci formation and soft agar assays. XTT assay showed that the cell growth rates in CRNN-expressing clones were significantly inhibited compared with control cells (*P*<0.01) (Figure 2B). Foci formation assay showed that the frequency of foci formation was significantly inhibited in CRNN-expressing clones compared with control cells (*P*<0.05) (Figure 2C). A similar result was obtained from soft agar assay, in which the colony formation in soft agar was significantly inhibited in CRNN-expressing clones compared with control cells (*P*<0.01 for CRNN-30 and *P*<0.05 for CRNN-180) (Figure 2D).

### CRNN inhibits tumor formation in nude mice

**Figure 3:**
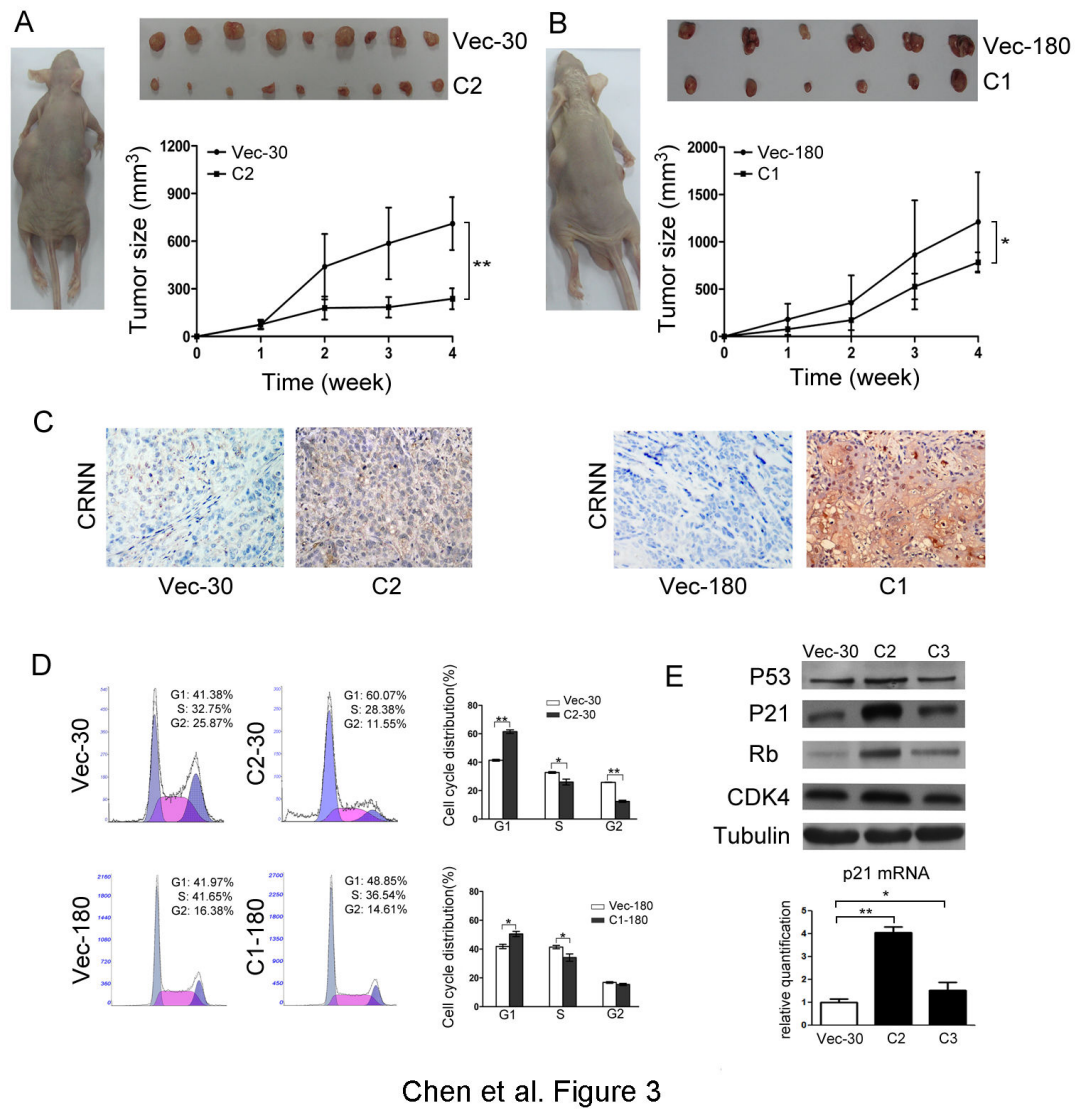
CRNN inhibits tumor formation in nude mice. (A) Representatives of tumors formed in nude mice induced by vector-transfected KYSE30 cells (left) and *CRNN*-transfected cells (right), respectively. Representatives of tumors derived from tested animals were shown in the upper panel. Tumor volumes were summarized in the lower panel. *, *P*<0.05; **, *P*<0.01. (B) Similar results were obtained in CRNN-transfected KYSE180 cells compared with vector controls. *, *P*<0.05; **, *P*<0.01. (C) IHC staining was performed to confirm the expression of CRNN in the tumors induced by *CRNN*-transfected cells but not in the tumors induced by vector-transfected cells. Original magnification, 200×magnification. (D) Representative and summary of DNA content detected by flow cytometry. The percentage of cells in S phase was significantly decreased in *CRNN*-transfected cells compared with control cells. *, *P*<0.05; **, P<0.01. (E) Western blot analysis shows that CRNN could upregulate P21^WAF1/CIP1^ and Rb expression significantly. P53 also increased slightly in CRNN-transfected cells. Tubulin was used as a loading control. *p21* mRNA level was determined by qRT-PCR. *, *P*<0.05; **, P<0.01.

To further explore the *in vivo* tumor suppressive ability of CRNN, tumor formation in nude mice was carried out by the injection of CRNN-C2 (KYSE30), CRNN-C1 (KYSE180), whereas Vec-30 and Vec-180 were used as controls. The results showed that tumor formation in nude mice was significantly inhibited in CRNN-expressing cells (*P*<0.01 for CRNN-30 and *P*<0.05 for CRNN-180) (Figure 3A and 3B). With immunohistochemical staining using anti-CRNN antibody, we confirmed that CRNN expression was re-established in CRNN-C2 or CRNN-C1-induced tumors (Figure 3C).

### CRNN arrests cell cycle at G1/S transition

To elucidate the mechanism underlying growth inhibition by CRNN, flow cytometry was used to compare cell distribution in cell cycle between CRNN-transfectants and control cells. The percentage of CRNN-30 in G0/G1 phases was significantly increased (*P*<0.01), whereas the percentage in S-phase was significantly decreased (*P*<0.05), compared with that in control cells, suggesting that CRNN was able to arrest cell cycle at G1/S phase (Figure 3D). Similar results were also observed in CRNN-180 cells (Figure 3D), which is consistent with the previous report [17]. To further reveal the potential molecular mechanism of CRNN in cell cycle arrest, the effects of CRNN on key cell cycle regulators P53, P21^WAF1/CIP1^ and Rb were tested. The result showed that expressions of P21^WAF1/CIP1^, Rb were upregulated significantly in CRNN-transfected cells compared with control cells (Figure 3E). P53 was also upregulated slightly in CRNN overexpressed cells. However, no significant difference was detected for CDK4 in this study. The mRNA level of *p21* also increased in CRNN overexpressed cells.

To test whether CRNN expression could induce a “differentiated state” of cells, we investigated keratin-4, an epidermal differentiation marker, in the CRNN overexpressing cells. No staining was observed in both CRNN overexpression and vector control cells (Figure S1).

### Knockdown of CRNN inhibits its tumor suppressive ability

**Figure 4:**
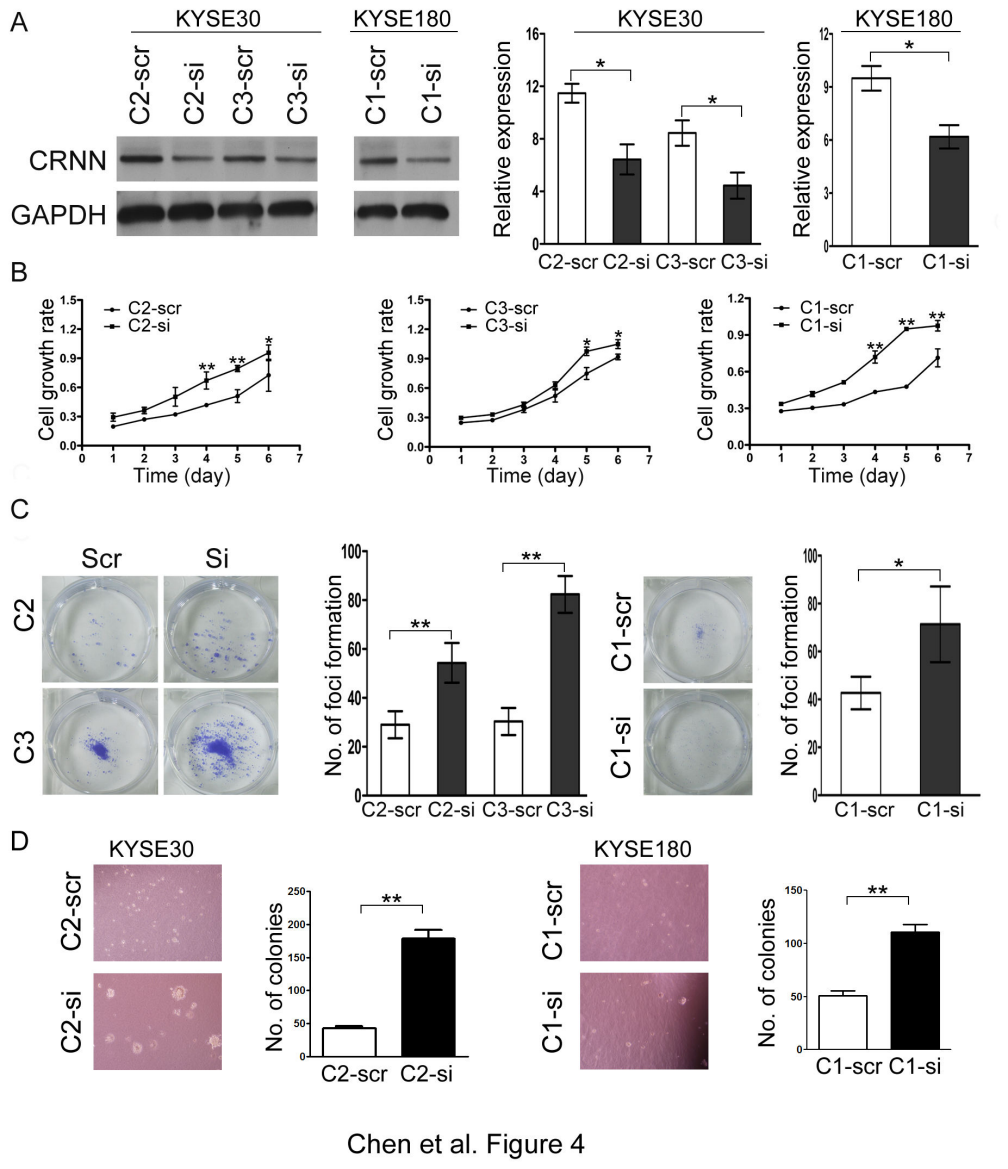
Silencing CRNN expression increases tumorigenicity. (A) Expression of CRNN was decreased by siRNA against CRNN compared with scramble control cells detected by western blot analysis (left panel). Fold change of CRNN expression was calculated from siRNA-CRNN relative to the scramble control (right panel). *, *P*<0.05. (B) Growth curve of CRNN-transfected cells treated with siRNA-CRNN were compared with scramble control cells by XTT assay. The results are summarized from three independent experiments. *, *P*<0.05; **, *P*<0.01. (C) Representatives of foci formation induced by cells treated with siRNA-CRNN, compared with scramble control treated cells. The number of foci was calculated and summarized in bar chart. The results are summarized from three independent experiments *, *P*<0.05; **, P<0.01. (D) Representatives of colonies formed in soft-agar in cells treated with siRNA and scramble control. The results are summarized from three independent experiments. **, P<0.01.

**Figure 5:**
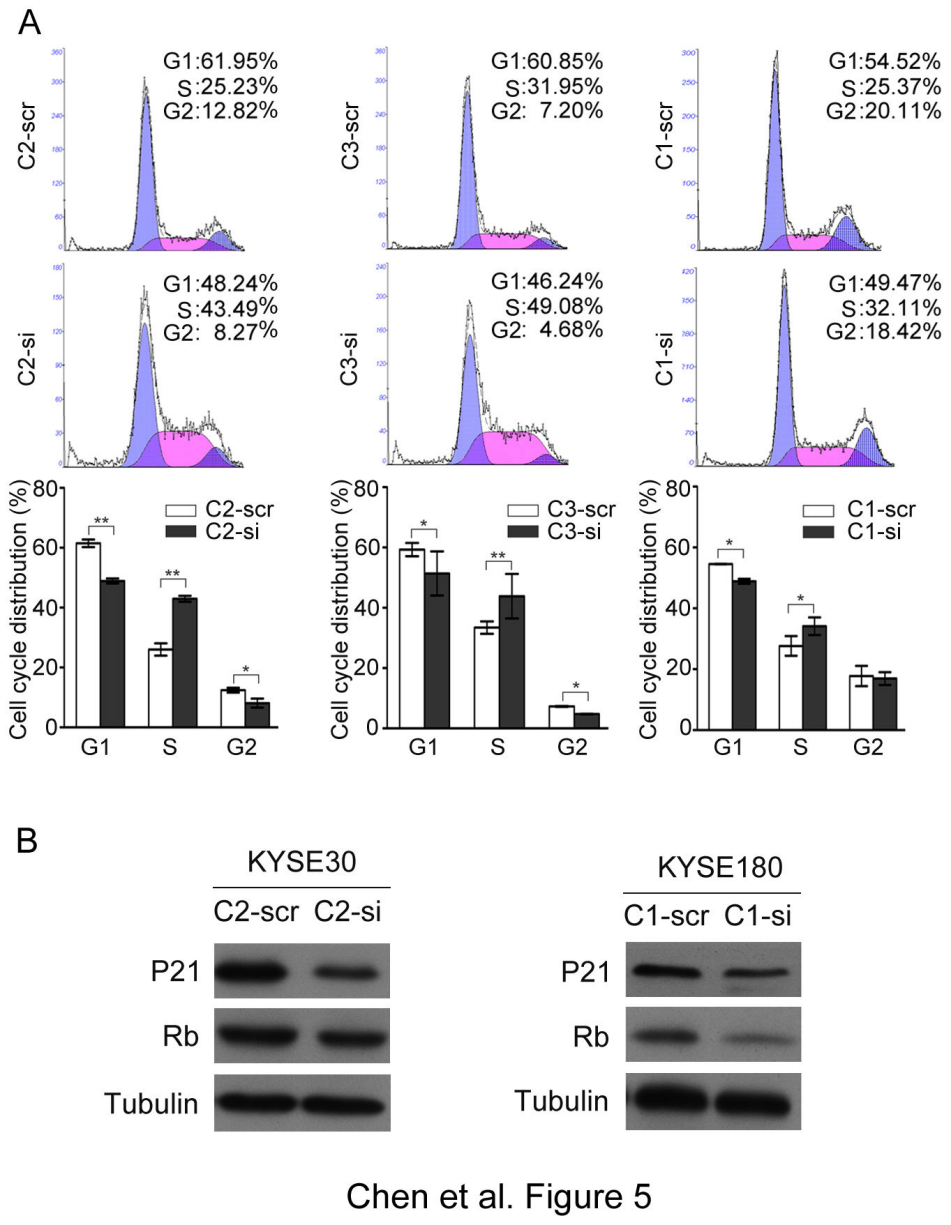
Silencing CRNN expression increases improper G1/S transition. (**A**) Cell distribution in cell cycle between siRNA-CRNN treated and scramble control cells. The results are summarized from three independent experiments (lower panel). *, *P*<0.05; **, *P*<0.01. (B) Expressions of P21^WAF1/CIP1^ and Rb expression in siRNA treated and scramble control cells were compared by western blot analysis. Tubulin was used as a loading control.

Expression of CRNN in CRNN-transfectants was silenced by RNAi with two siRNAs targeting CRNN. Western blotting result showed that CRNN expression could be effectively silenced (Figure 4A). Cell growth assay demonstrated that the cell growth rate was significantly increased in siCRNN-treated cells compared with scramble-treated cells (*P*<0.05) (Figure 4B). Similarly, foci formation assay revealed that the frequency of foci formation was significantly increased in siCRNN-treated cells compared with scramble-treated cells (Figure 4C). Soft-agar assay results demonstrated that the number of colonies formed in soft agar increased in CRNN knock-down cells compared with scramble control cells (Figure 4D). Furthermore, DNA content analysis by flow cytometry showed that silencing CRNN expression was able to increase the G1/S transition. The percentage of cells in the S phase was significantly increased in siCRNN-treated cells compared with scramble-treated cells (*P*<0.05) (Figure 5A). Western blot analysis showed that p21^WAF1/CIP1^ and Rb were downregulated in siCRNN-treated cells compared with scramble-treated cells (Figure 5B).

## Discussion

CRNN has been proposed as a squamous-cell specific gene because of its expression in esophageal tissue, cervical squamous epithelium, and murine skin, and absence of expression in another 15 glandular tissues [18,19]. Downregulation of CRNN was reported in some cancers, including esophageal cancer [6–10], oral squamous cell carcinoma [17], and head and neck squamous cell carcinoma [20]. However, the molecular mechanism of CRNN in tumor remains unclear. In the present study, we found that CRNN was downregulated in 55.02% of primary ESCC tumors, which was significantly associated with advanced clinical stage (*P*=0.039), lymph node metastases (*P*=0.027) and poor survival of patients with ESCC (*P*<0.001). Multivariable analyses showed that the downregulation of CRNN could be used as an independent prognostic predictor for ESCC patients. Furthermore, we investigated the mechanisms underlying CRNN downregulation in ESCC cells. However, neither hypermethylation nor histone modification was associated with CRNN downregulation in ESCC (data not shown).

Loss of heterozygosity (LOH) at 1q21 region has been frequently detected in various solid tumors, including esophageal squamous cell carcinoma [21], breast cancer [22], insulinoma [23] and esophageal adenocarcinoma [24]. Interestingly, loss of 1q21 has been associated with tumor malignancy [23] and shorter overall survival [24]. To explore whether CRNN inactivation is correlated with single nucleotide variation in the promoter region of CRNN, a fragment (-2,000 to -1) from promoter region of CRNN was sequenced in 10 ESCC cases. Four known SNPs were found in this promoter region. After systematical analysis, none of them was significantly associated with CRNN downregulation (data not shown). Another possible mechanism might be involved in the CRNN inactivation, micro-RNA regulation [25], was not investigated in the present study.

CRNN gene is located on the chromosome 1q21, where thirteen S100 family members are tightly clustered [26,27]. The functions of S100 genes are very complex and they play different roles in cancer development and progression [28]. For example, S100A4, S100A6, S100A7 and S100B play oncogenic roles in cancer development [29,30], whereasS100B, S100A2, and S100A11 are believed as TSGs [31–33]. In the present study, tumor suppressive function of CRNN was characterized by both *in vitro* and *in vivo* assays including cell growth, foci formation and soft agar assays, and tumor formation in nude mice. The results demonstrated that CRNN could effectively suppress cell growth, foci formation and colony formation in soft agar, and inhibit tumor formation in nude mice. A further study revealed that CRNN was able to inhibit G1/S transition through the upregulation of P21^WAF1/CIP1^ and Rb. G1/S phase transition is a major checkpoint for cell cycle progression and P21^WAF1/CIP1^ is one of the critical negative regulators during this transition [34,35]. Rb is another important tumor suppressor in the cancer development. When it is mutated or deleted, E2F transcription factor would be released and induce the expression of genes that stimulate cell growth [36,37]. We further tested P53 protein level and found that P53 increased slightly in CRNN overexpressed cells compared with vector control cells. It was reported that p53 could be activated by DNA damage in KYSE30 with mutant p53 as other ESCC cell line with wide-type p53 [38]. qRT-PCR results also indicated that *p21* mRNA level increased in CRNN overexpressed cells. Taken together, we hypothesize that overexpression of CRNN could upregulate P53, and it subsequently increases P21 and Rb and inhibit G1/S transition.

In the present study, the tumor suppressive function of CRNN has been clearly demonstrated, however, the molecular mechanism of CRNN in ESCC development remains unclear. CRNN has been reported to allow cells to tolerate normally lethal levels of deoxycholic acid and protect from the toxic effect of bile acid as a survival factor [39]. Further study identified CRNN as a potential component of epithelial immunity based on its strong signature of adaptive evolution on DNA sequence of a type that is commonly associated with a coevolutionary arms race with a pathogen [40]. Therefore, the expression of CRNN protein will presumably help maintain the barrier function in squamous epithelium in response to injury and function as a tumor suppressor [10]. In conclusion, we demonstrate that CRNN is a potential tumor suppressor in ESCC via arresting cell cycle progression at G1/S checkpoint by upregulating P21^WAF1/CIP1^ and Rb. A better understanding of the tumor suppressive role of CRNN will significantly improve our knowledge in the development of ESCC, and may lead to a more effective management of ESCC patients with the inactivation of CRNN.

## Supporting Information

Figure S1 **IHC staining with CK4 and CK (pan) in CRNN overexpression cells and vector control cells.** Original magnification: 200× magnification.
(JPG)
