# Supplementary figures and images for: Characterization of Tumor Suppressive Function of cornulin in Esophageal Squamous Cell Carcinoma
Source: PLoS One. 2013 Jul 24;8(7):e68838. doi: 10.1371/journal.pone.0068838 (PMC3722219; doi:10.1371/journal.pone.0068838)

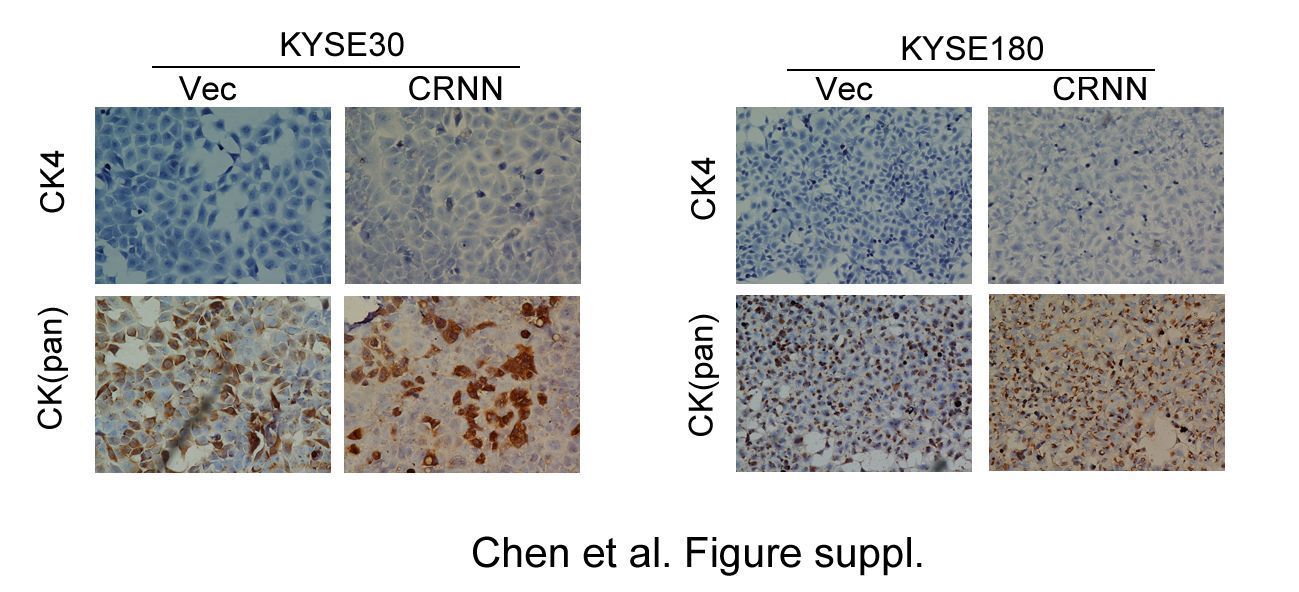

Supplement: Figure S1 — IHC staining with CK4 and CK (pan) in CRNN overexpression cells and vector control cells. Original magnification: 200× magnification. (JPG) [file pone.0068838.s001.jpg]
